# Prevalence and Associated Factors of Common Mental Disorders in Women: A Systematic Review

**DOI:** 10.3389/phrs.2021.1604234

**Published:** 2021-08-23

**Authors:** Héllyda de Souza Bezerra, Roberta M. Alves, Aryelly Dayanne d. Nunes, Isabelle R. Barbosa

**Affiliations:** Graduate Program in Public Health, Federal University of Rio Grande do Norte, Natal, Brazil

**Keywords:** mental health, systematic review, women, prevalence, mental disorders

## Abstract

**Objectives:** To identify the prevalence and factors associated with common mental disorders in adult women.

**Methods:** Searches were carried out in the PubMed, Web of Science, Science Direct, Scopus, Cinahl, Google Scholar and Open Gray databases. The study protocol was registered with PROSPERO under number CRD42020168231. Cross-sectional studies showing the prevalence of common mental disorders in women over 18 years were included. Studies with men, children and pregnant women of another age group and with other mental disorders and other types of studies were excluded. The Joanna Briggs Institute checklist was used to assess the risk of bias.

**Results:** Nineteen studies were included in this review. The prevalence of CMD ranged from 9.6% to 69.3%. The main associated factors were unemployment, indebtedness, low income, being a housewife, smoking, low education, poor self-rated health, being single, divorced or widowed. The risk of bias in the studies was classified as low and moderate.

**Conclusion:** This review revealed a variable prevalence rate of CMD in adult women. Public policies are needed to create strategies to prevent the mental illness of these women.

## Introduction

Mental disorders are a serious public health problem affecting low, middle and high income countries all over the world, accounting for about one third of mental illness globally. An estimated 322 million people are diagnosed with mental disorders worldwide. In Brazil, 9.3% of the population have symptoms related to anxiety and 5.8% have depression [1, 2].

Common Mental Disorders (CMD) have anxiety, depression and suicidal ideation as main symptoms of psychological distress, in addition to non-psychotic symptoms such as irritability, insomnia, fatigue and memory problems. However, CMD do not always meet the formal criteria of the international classification of diseases (ICD-10) or the Diagnostic and Statistical Manual of Mental Disorders (DSM-V) [3, 4].

Common mental disorders affect the activities of daily living, damaging social, family and work environment relationships. In addition, in the case of young adults, they cause socioeconomic vulnerability, decreasing productive capacity and leading to social isolation and increased use of health services [5].

One in five people may to develop CMD during their lifetime, and the probability is higher in the female sex [6]. This can be explained by socio-cultural factors linked to the female sex, which is more exposed to domestic work overload, domestic violence, domestic stressors, and multiple roles developed in society, both in the home and work contexts [7].

In addition to socio-cultural factors, women present biological vulnerability to CMD symptoms particularly linked to the reproductive period. The role of estrogen in mood modulation can explain, in part, the high prevalence of mood and anxiety disorders in women from menarche to menopause. Besides the association between hormonal variation during premenstrual, puerperium and menopause periods and depressive mood, the hormones present in oral contraceptives and hormone replacement therapy also influence mood [8]. The higher prevalence of CMD in women is also associated with the greater detection of symptoms and search for health services in this sex [9].

The psychological suffering generated by CMD in adult women triggers a lower quality of life and difficulty in developing activities of daily living. Despite the understanding about the population segments most vulnerable to CMD, research on mental health, specifically of adult women, is still scarce [10]. Identifying the magnitude of this problem and the most vulnerable groups allows proposing public policies aimed at women’s health and planning more effective health actions to prevent these disorders, as well as encouraging multidisciplinary interventions to adequately treat women who have CMD.

Besides being essential to know the prevalence of CMD in adult women, it is also necessary to know the factors associated with these disorders, so that they can be prevented and the number of patients with CMD can be minimized. The goal of this study was to identify the prevalence and associated factors of CMD in adult women. The following research question was proposed: “What is the prevalence of CMD in adult women and what are the associated factors?”.

## Methods

### Protocol and Registration

To start this systematic review, a protocol was created and registered in the International Prospective Register of Systematic Reviews (PROSPERO) (CRD42020168231). The Preferred reporting items for systematic reviews and meta-analyses (PRISMA) statement [11] was used as a guide to write this review.

### Eligibility Criteria

Cross-sectional studies reporting data on prevalence of CMD and associated factors in women over 18 years were included. Studies with the following characteristics were excluded: 1) conducted with men, children and pregnant women; 2) conducted with women under 18; 3) addressing other mental disorders and/or diseases; 4) not presenting prevalence rates and/or associated factors, or insufficient data for calculations; 5) cohort studies, case-control studies, diagnostic studies, clinical trials, reviews, letter to the editor, conference abstracts, and opinion articles.

### Sources of Information

Searches were conducted in the PubMed, Web of Science, Science Direct, Scopus, Cinahl databases, in addition to the Google Scholar and Open Gray. No restrictions as to language or publication date were adopted. A librarian was consulted during the search strategy in the bibliographic bases. The MeSH terms and key words “Women” and “Mental Disorders,” “Women’s Groups” and “behavior disorders” and the key word “common mental disorders” were used in the search (Supplementary Appendix SA). Experts were consulted *via* e-mail to find more references.

The results obtained in the database search were inserted in the reference manager Mendeley Desktop® version 1.19.4 for the elimination of duplicates. Then, the resulting articles were transferred to Rayyan QCRI® software to be sorted by titles and abstracts. Searches were carried out on January 12, 2020.

### Study Selection

Titles and abstracts were read and those that met the eligibility criteria were read in full length. All steps were performed by two independent researchers (HSB and RMA). In both stages, meetings were held to resolve disagreements and reach consensus; there was no need to consult the third reviewer (IRB).

After the initial screening, the results obtained were submitted to complete textual reading in the Rayyan QCRI® used ensure blindness during the research, based on the eligibility criteria. After this analysis, studies were selected to be included in the systematic review.

### Data Extraction

The data collected from the studies selected by the first and second authors independently (HSB and RMA) were: authors, year of publication, city, country, sample size, age of participants, prevalence of CMD, methods, instruments for identifying CMD, and associated factors in the adjusted analysis. The types of associated factors were not selected beforehand, but were inserted as they were found in the studies. In both stages, disagreements were resolved through consensus meetings; there was no need to consult the third reviewer (IRB).

### Risk of Bias

The methodological quality of the included studies was evaluated by the Checklist for Analytical Cross Sectional Studies from The Joanna Briggs Institute [12].

The eight questions were answered by the first and second reviewers independently with “Yes,” “No,” “Unclear” or “Not applicable.” When necessary, a consensus meeting was held. The risk of bias results was classified into 1) low risk, if the studies reached more than 70% of “yes” scores; 2) moderate risk, if the studies reached between 50 and 69% of “yes” scores; and 3) high risk, if the studies reached less than 49% “yes” scores [13]. The graphical illustration of risk of bias was created using the Review Manager 5.3 software (RevMan 5.3, The Nordic Cochrane Center, Copenhagen, Denmark).

## Results

### Search Results

**FIGURE 1:**
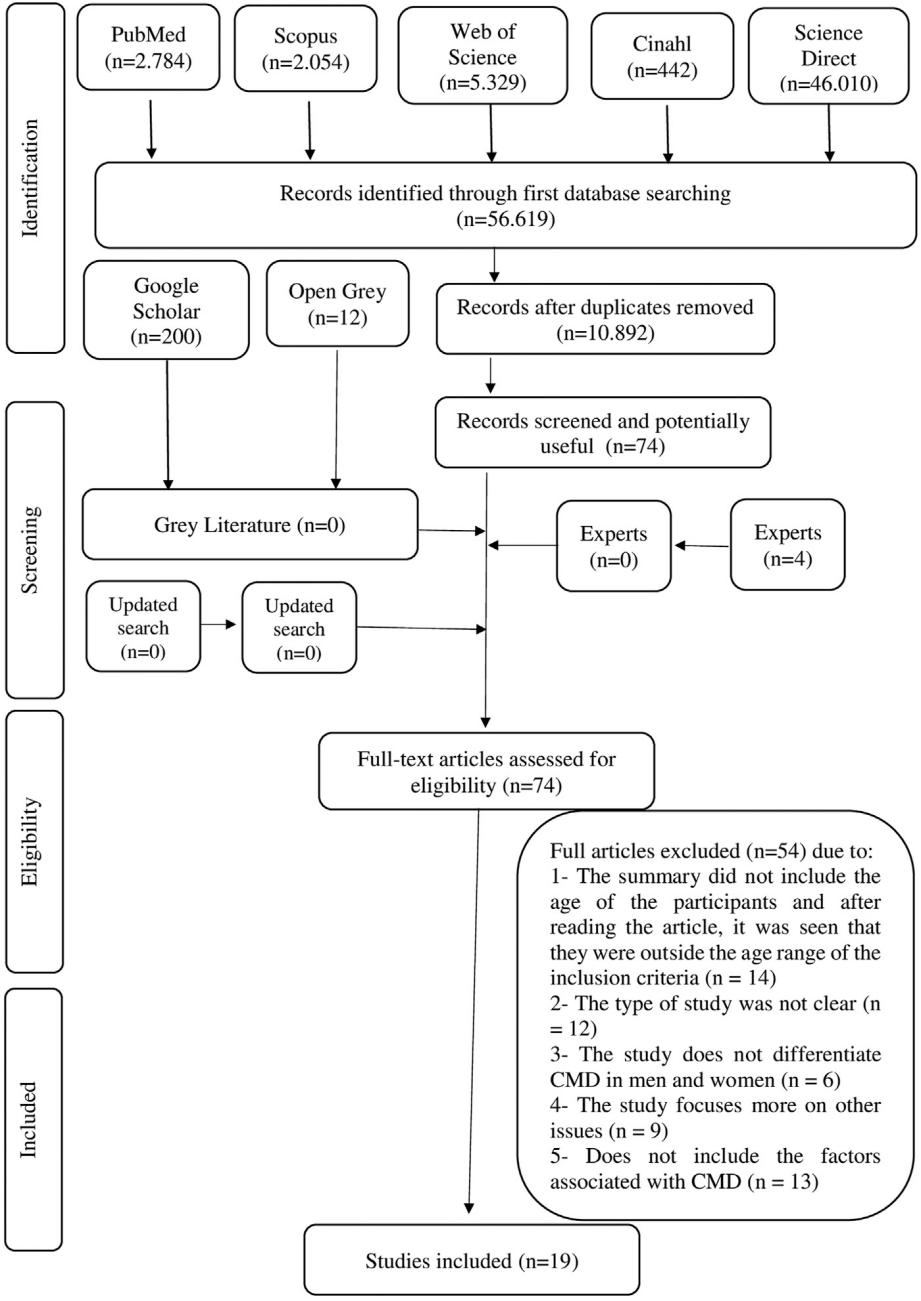
Flowchart of article selection. Prevalence and associated factors of common mental disorders in women: a systematic review, 2020. * Adapted from PRISMA.

A total of 56,619 articles were found in the searches. After removing the duplicates, 10,892 articles remained for reading of titles and abstracts. In the screening phase, the titles and abstracts were read, and those that did not meet the eligibility criteria were subsequently excluded. The main exclusion criteria were related to the participants’ age (exclusion criteria 1 and 2). Therefore, 74 articles were read in full length; and of these, 54 were excluded (Supplementary Appendix SB) following the same criteria. No study from the gray literature was included. There was no reply from the experts contacted. Thus, 19 articles were included in this systematic review (Figure 1).

### Characteristics of the Included Studies

Publication year of the study: the included studies (Table 1) were published from 2004 [14] to 2018 [15–18].
Study site: The articles included several countries, spread over four continents. Of the 19 articles, eight were developed in South American countries: Brazil [6, 15–20] and Colombia [21]; one was developed in North America: United States [22]; six were developed in Europe: Belgium [14], Italy [23], France [24], Greece [25], Spain [26], Norway [27]; two were developed in Asia: Qatar [24], India [28]; and two were developed in Africa: Ghana [29] and Kenya [30].
Sample size: the studies presented different sample sizes, varying from 234 [6] to 45,399 women [27].
Participants’ age: seven studies included women over 18 years old, without specifying the age group, five studies included women up to 59 years old, and seven studies included elderly women (over 60 years old). The studies by Rocha et al. [16] and Rocha et al. [17] included women up to 82 years old, with a mean age of 38.96 years. In the studies by Duran et al. [22] and Borges et al. [6], the included participants had an average age of 27.8 and 45, respectively, being the lowest and highest averages found in the studies analyzed. The majority of the studies did not include elderly women.
Prevalence of CMD: nine articles presented the prevalence for each of the disorders, such as mood disorders, minor depressive disorders, anxiety and some phobias [14, 18, 20, 22, 23, 25, 27, 31] and 10 presented a single prevalence value for all CMD [6, 15–17, 19–21, 26, 28, 30] The prevalence of CMD varied from 9.6% [26] to 69.3% [19].
The instruments used to identify CMD: As for the instruments used to identify CMD, there was variation among the instruments used. The most used instrument (seven studies) [6, 15–17, 20, 28, 30] was the Self-Reporting Questionnaire (SRQ-20). The others used other instruments to screen for mental disorders: Primary Care Evaluation of Mental Disorders (PRIME-MD) [14], Mini-International Neuropsychiatric Interview (MINI) [24], General Health Questionnaire (GHQ-12) [19, 21], the Composite International Diagnostic Interview (CIDI) [23], the Gatekeeper (GPs) for health services [27], and the Kessler 6 (K6) [26, 29].
Factors associated with CMD: The studies identified an association between CMD and unemployment [15–17, 20, 23, 26], having a large number of debts [22, 30], being a housewife [23, 26], being a smoker [15, 21, 26], having a lower educational level [22, 27, 31], having poor self-rated health [22, 28] being single [19] divorced [25, 27] or widow [27], and having low income [19, 20, 31]. Leisure activities were a protective factor against CMD [16]. Two studies showed no associated measures. Accordingly, associated factors were presented in 17 studies.

**TABLE 1:** Characteristics of the included studies. Prevalence and associated factors of common mental disorders in women: a systematic review, 2020.

Authors	City/Country	Age in years (mean)	Sample	Method for identification of CMD	Prevalence of CMD	Associated factors (95% CI)
Ansseaua et al. [14]	Belgium	Over 18	1,360	Questionnaire for mental disorders (PRIME-MD)	MD: 35.3%	*
					MDD: 3.8%	
					AD: 21.9%	
Duran et al. [22]	United States	18 to 45 (mean 27.8)	234	Questionnaire for mental disorders (GHQ-12)	MD: 44%	Debts: PR = 1.5 (95% CI = 1.1–2.1)
						Poor self-rated health: PR = 1.5 (95% CI = 1.2–2.0)
					AD: 58%	Debts PR = 1.4 (95% CI = 1 .1–1.9)
						Low educational level (below high school) PR = 1.4 (95% CI = 1. 1–1.7); Poor self-rated health: PR = 1.4 (95% CI = 1.1–1.6)
Girolamo et al. [23]	Italy	Over 18	2,391	Questionnaire for mental disorders (CIDI)	Prevalence of any mental disorder: 24.4%%	MD
						Previously married: OR = 2.0 (95% CI = 1.2–3.4)
						Unemployed: OR = 2.1 (95% CI = 1.1–4.2)
					Prevalence of MD: 14.9%	AD
						Housewife: OR = 2.1 (95% CI = 1.2–4.0)
					Prevalence of AD: 16.2%	CMD
						Housewife: OR = 1.9 (95% CI = 1.1–3.1)
Cohidon et al. [24]	France	Over 18	21,337	Mini-International Neuropsychiatric Interview (MINI)	MD: 16%	*
					AD: 25%	
Campo-Arias et al. [21]	Colombia	18 to 65 (mean 38.3)	1,740	Questionnaire for mental disorders (GHQ-12)	CMD: 15.7%	Alcohol abuse: OR = 6 .4 (95% CI = 2.7–15.2)
						Daily smoking: OR = 3.3 (95% CI = 2.1–5.0)
						Chronic diseases: OR = 2.0 (95% CI = 1.4–2.8)
						Daily coffee consumption: OR = 1.3 (95% CI = 1.0–1.8)
Fortes et al. [19]	Brazil	18 to 65	327	Questionnaire for mental disorders (GHQ-12)	CMD in extremely poor women: 69.35	Single marital status: OR = 1.23 (95% CI = 1.08–1.40), Poverty level: family income per capita less than US $ 40 per month: OR = 1.08 (95% CI = 0.95–1.24) and extremely poor women
					CMD in women who are not extremely poor: 56.78%	
Ghuloum et al. [31]	Qatar	18 to 65	893	Self-administered questionnaire validated by the authors	CDM: 55.6%	CMD
					MDD: 13.3	Age group, marital status, education, occupation, and monthly family income (*p* < 0.001)
					AD: 10.9%	
					Phobia: 6.6%	
Jansen et al. [20]	Brazil	18 to 24	880	Screening questionnaire for common mental disorders (SRQ-20) and Quality of Life Questionnaire (SF-36)	CDM: 32.2%	CMD
						Belonging to economic class D or E: OR = 2.11 (95% CI = 1.67–2.66)
						Unemployment: OR = 1.55 (95% CI = 1.24–1.94)
						General health status: OR = 17.6 (95% CI = 15.4–19.7)
Menil et al. [29]	Ghana	Over 18 years (mean 37)	2,814	Questionnaire for mental disorders (Kessler 6 - K6)	This study failed to estimate the prevalence of CMD because the K6 tool, commonly used for this purpose, has not yet been indexed in Ghana. The average score found on K6 was 27.1	CMD
						Low levels of education, poverty and unemployment (*p* = 0.001, 0.02, 0.0001, respectively)
Skapinakis et al. [25]	Greece	18 to 70 (mean 42 ± 15 SD)	2,466	Questionnaire for mental disorders (CIS-R)	MDD: 3.68%	Depression
					AD: 8.45%	Separated: OR = 2.10 (95% CI = 1.13 –3.90)
					All phobias: 3.64%	Widowed: OR = 2.44 (95% CI = 1.39–4.30)
						Unemployed OR = 2.46 (95% CI = 1.20–5.04)
						AD
						Divorced: OR = 1.89 (95% CI = 1.22–2.93)
						Widowed: OR = 1.66 (95% CI = 1.05–2.63)
Jurado et al. [26]	Spain	18 to 50	906	Questionnaire for mental disorders (Kessler 6–K6)	CDM: 9.6% in Spanish women	Divorced or widowed: OR = 2.1 (95% CI = 1.3–3.5)
						Unemployed or housewife: OR = 0.59 (95% CI = 0.35–1.0)
					CDM: 24.9% in migrant women	Daily smoking: OR = 1.4 (95% CI = 0.97–2.2)
						Being a migrant: OR = 3.1 (95% CI = 2.1–4.6)
Borges [6]	Brazil	Over 18 (mean 45)	365	Screening questionnaire for common mental disorders (SRQ-20)	CDM: 44.1%	Lower quality of life in the domains: physical (*p* < 0.001), psychological (*p* = 0.018), and social relationships (*p* = 0.001)
Soni et al. [28]	India	18 to 45	658	Screening questionnaire for common mental disorders (SRQ-20)	CMD: 22.8%	Report of a worse health state: OR = 9.34 (95% CI = 5.93–14.70) and Health income expenditure: OR = 2.25 (95% CI = 1.48 to 3.44)
						Increase in the number of consultations in the previous year: OR = 1.22 (95% CI = 1.05–1.42)
Gjesdal et al. [27]	Norway	18 to 67	45,399	Gatekeeper (GPs) model for healthcare	Depression: 43%	Low education (high school): OR = 0.90 (95% CI = 0.85–0.95)
					Anxiety: 23%	
					Stress: 75%	
					Sleep problems	Divorced: OR = 1.17 (95% CI = 1.10–1.24)
					All: 47%	
Husain et al. [30]	Kenya	Over18	429	Screening questionnaire for common mental disorders (SRQ-20)	CDM: 20%	Financial difficulties: OR = 4.3 (95% CI = 2.5–7.3)
						Relationship difficulties: OR = 1.4 (95% CI = 0.6–3, 4)
Audi et al. [15]	Brazil	Over 18 (mean 30.8 ± 9.6 SD)	1,013	Screening questionnaire for common mental disorders (SRQ-20)	CDM: 66.7%	Unemployment: OR = 1.37 (95% CI = 1.04–1.80)
						Smoking: OR = 1.58 (95% CI = 1.18–2.11)
						Physical inactivity: OR = 1.56 (95% CI = 1.19–2.13)
						Violence witnessed in the family during childhood or adolescence: OR = 1.40 (95% CI = 1.05–1.86)
Santos et al. [18]	Brazil	20 to 59	369	Screening questionnaire for common mental disorders (SRQ-20) and assessment of intimate partner violence (Conflict Tactic Scales -CTS-2)	Symptoms of depressed and anxious mood: 79.4%	Symptoms of depressed and anxious mood
					Somatic symptoms: 78.8%	Physical abuse without sequelae (minor): OR = 1.82 (95% CI = 1.01–3.30)
					Symptoms of depressive thoughts: 78.8%	Physical abuse without sequelae (severe): OR = 2.58 (95% CI = 1.06–6.25)
					* The authors calculated from the results of the study	Physical abuse with sequelae (minor): OR = 3.7 (95% CI = 1.29–10.63)
Rocha et al. [16]	Brazil	19 to 82 (mean 38.96)	2099	Screening questionnaire for common mental disorders (SRQ-20)	CDM: 22.7%	Unemployment: OR = 1.13 (95% CI = 1.08–1.18)
Rocha et al. [17]	Brazil	19 to 82 (mean 38.96)	2099	Screening questionnaire for common mental disorders (SRQ-20)	CMD: 22.7%	Leisure: OR = 0.50 (95% CI = 950.35–0.72)

Legend: * There are no association values; CI, confidence interval; OR, odds ratio; CMD, common mental disorders; MD, mood disorders; MDD, minor depressive disorders; AD, anxiety disorders.

### Risk of Bias

**FIGURE 2:**
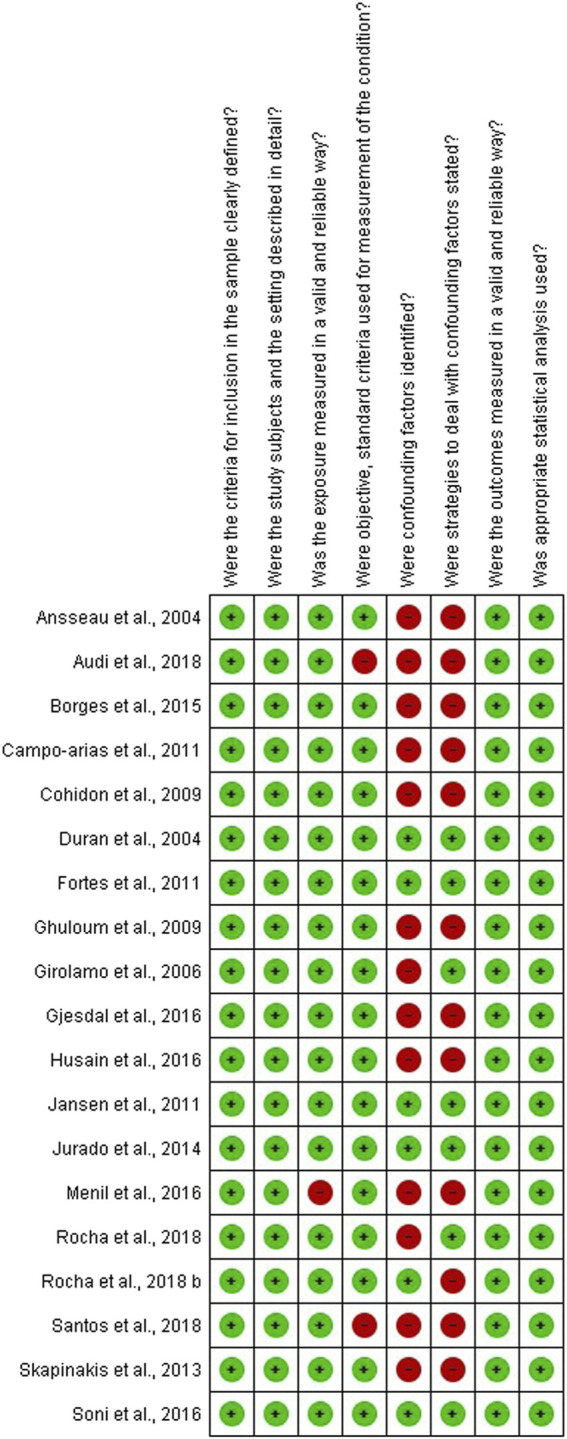
Graphical illustration of the summary of the risk of bias. Prevalence and associated factors of common mental disorders in women: a systematic review, 2020.

Regarding the risk of bias, the studies were classified as having low [6, 14, 16, 17, 19–28, 31] and moderate [15, 18, 29] risk (Supplementary Appendix SC). In the majority of the studies, the items “were confounding factors identified” and “were strategies to deal with confounding factors stated” were scored as presenting a high risk of bias (Figure 2).

## Discussion

The objective of this systematic review was to identify the prevalence and associated factors of CMD in adult women. The prevalence of CMD ranged from 9.6 to 69.3% and was associated with individual factors and the socioeconomic and work context. Twelve studies had a low risk of bias, showing the good methodological quality of the included studies.

There are several questionnaires for screening and diagnosing mental disorders. Obtaining good results with the use of these questionnaires involves several factors, such as their reliability, validity and high sensitivity. The questionnaires must be short, brief and of easy application [32]. Another factor that directly affects the result is the training of the people who will apply these instruments. The use of the instrument and its proper application improves the capacity to detect CMD. Their inappropriate use, on the other hand, can lead to inadequate screening, underestimating or decreasing the prevalence of CMD [33]. Different instruments used in populations with varied culture, economics and linguistic backgrounds can generate variations in the prevalence of CMD [32], as seen in the present systematic review.

As for the samples, there was a variety, but most studies had probabilistic samples, maintaining the assumptions of internal validity. Therefore, despite the variety of samples, all included studies have representative samples to estimate the prevalence of CMD in adult women. As for the age group of the women, the average ages corresponded to young adult women. The study by Souza et al. [34] showed similar results in their research, related to the disadvantages lived by younger women because they often have a double workload as they work and care for domestic tasks. The women who work and carry this family burden generally renounce their own care in order to take care of the family. This ultimately leads to anxiety, frustration, anguish and mental illness.

As for the study site, this systematic review included countries from 04 continents (America, Europe, Africa and Asia), demonstrating a good geographic representation and worldwide impact. CMD present a growing increase in numbers worldwide. Africa and America stand out as the continents with the highest prevalence of women with depression. America also has the world’s highest prevalence of women with anxiety. Regions with lower socioeconomic conditions show worse health conditions and increased rates of CMD [2, 3]. The study by Jurado et al. [26] identified that the lowest prevalence of CMD (9.6%) occurred in women from Spain. Compared to Spanish women, the chances of CMD were higher for Latin American, Moroccan and other African migrant women. The study on the global burden of mental illness including 28 countries around the world showed that the prevalence of all mental disorders in the past 12 months was higher in Latin American countries than in Spain and other European countries [35]. The low prevalence of CMD found in this systematic review may be explained by the fact that most women with mental disorders in Spain are able to receive primary health care, as well as by the high consumption of psychotropics by women in this country [36].

Latin America was the continent with the largest number of studies in the sample of the present review. In the last few years, mental disorders make up almost a quarter of the existing diseases in this continent, due to the lack of access to treatments and to follow-up [37]. Brazil is the Latin American country with the highest prevalence of people with depression and anxiety in the world, with a high prevalence among women [2]. The study by Fortes et al. [19] conducted in Brazil in the city of Petrópolis/RJ was the one with the highest prevalence of CMD, with a rate of 69.3% in extremely poor women. This high prevalence rate in Brazil can be explained by multiple factors, such as adverse socioeconomic conditions, high unemployment rates, low education levels, and generalized urban violence [38].

There was considerable variation in the prevalence of mental disorders worldwide and these results should reflect different social and cultural factors of the women included in each study. Steel et al. [3] showed that the global prevalence of CMD was 17.6%, for an adult in the last 12 months, and 29.2%, throughout life. Different causes may be associated, however, with the highest rates of CMD in continents and countries with precarious conditions of access to health [2]. There are factors that are linked to the particularity of each studied country and/or region, such as cultural, economic and social factors, thus interfering directly in mental health care, being responsible for the increase or reduction of CMD in adult women.

The variation in the prevalence rates of CMD presented between studies may be related to differences in the characteristics of the populations studied (race and ethnicity, age group, socio-cultural aspects) and to methodological issues, such as the different instruments used for screening and different points of cuts used for the same instrument.

As for the instruments used for screening CMD, the SRQ-20 was the most used. The study by Bolsoni and Zuardi [33] analyzed the reliability and/or internal consistency of brief instruments for tracking multiple mental disorders, and pointed out that the SRQ-20 was widely used in various cultural contexts. This questionnaire is validated in several countries and recommended by the World Health Organization for it meets the criteria in terms of ease of use and reduced cost [39, 40]. The sensitivity and specificity of this instrument is 83 and 80%, respectively, and the cut-off point for women is 7/8. Regarding the cut-off point, there is a considerable variation in the studies depending on the cultural and temporal context in which the instrument was applied; in some studies, the cut-off point is lower for men (5/6) than for women (7/8), while in others, the cut-off point is 7/8 for both sexes [8, 41].

The PRIME-MD was used only in the study by Ansseau et al. [14]. This is an instrument with good diagnostic performance in adults (sensitivity of 83%, specificity of 88%, and positive predictive value of 80% for the diagnosis of any psychiatric disorder). It assesses groups of CMD found in the general population, namely, mood disorders, anxiety, somatoform disorders, alcohol and eating disorders [42]. The Patient Health Questionnaire-9 (PHQ-9) was created from the PRIME-MD, and it is a more synthesized version, of easier application in adults [43].

With regard to the other questionnaires used for screening CMD, the MINI, the GHQ-12, the CIDI 2.1, and K6 scale are used in the adult population. They are easy and quick to administer, have satisfactory reliability and validity, and present sensitivity and specificity above 80% [44–47]. The study by Gjesdal et al. [27] used the Norwegian Gatekeeper (GP) model for screening CMD, in which general practitioners formulated the diagnosis at the end of the consultation based on the clinical evaluation.

The diversity of existing instruments to perform CMD screening and the high prevalence of CMD in women make it necessary to validate specific instruments for the female population, considering individual, social and cultural factors. Nevertheless, the included studies considered these factors and conducted a representation of different regions of the world, with low/moderate risk of bias.

As found in the present study as well as in others, multiple factors are associated with CMD in adult women [48–50], including unemployment [15, 16, 20, 23, 26], low schooling [22, 27, 31], and low income [19, 20, 31].

Low education is associated with CMD. According to some studies [49, 51], higher schooling generates better cognitive skills, informed decision-making, financial independence, better quality food, leisure and health, directly favoring better physical and mental health status. Low schooling makes women more prone to greater difficulty to find a job, lower salaries, and especially unemployment [52]. Unemployment, which is often caused by fewer years of study, generates financial difficulties and mental suffering [10]. Therefore, smaller number of years of schooling, unemployment and lack of income may be related to the development of CMD in women.

The variable “being a housewife” was a factor associated with CMD in some studies [23, 26]. Many women who are in the role of “housewife” feel devalued, have less autonomy and feel socioeconomically invisible. Furthermore, there is a lack of recognition of the family and society, as they are not within the current productive work standards. All these aspects together generate feelings typical of CMD [53].

Smoking was also a factor associated with CMD [15, 21, 26]. Although the use of cigarettes is more frequent among men, there is an association between use of cigarette and depression in women (He et al. 2014). Some studies [54–56] also found that lower income, low education and not having a partner are related to smoking. It is suggested that lower socioeconomic level can induce stress and this, in turn, is a trigger for the habit of smoking.

Being single [19], divorced [25, 27] and widowed [25] were factors associated with CMD in women. According to Senicato et al. [49], the presence of a partner and a good marital relationship generates social support, minimizing stress situations in the daily life. In marriage, individuals share a wide variety of activities, which include meals, housework, childcare, leisure and rest. The absence of a partner can lead to loneliness and psychological distress.

As for health assessment, two studies [22, 28] showed that women with CMD had a poor self-rated health. In general, women have other comorbidities associated with CMD, which can lead to loss of quality of life [49].

Violence against women [15, 18] was another factor associated with CMD. The study by Mendonça and Ludemir [57] showed that psychological, physical and sexual violence by an intimate partner was associated with CMD and the more severe the aggression, the greater the impact on the women’s mental health. The growth in domestic violence must be combated in order to minimize the symptoms of CMD and other mental disorders.

Rocha [17] showed that having leisure activities is a protective factor for CMD. As Araújo et al. [58] explain, leisure activities are important for people’s well-being and quality of life, reducing symptoms of sadness, depression and anxiety. Regular physical exercise also reduces symptoms of depression, insomnia and improves attention span [59]. Thus, it is observed that the implementation of leisure time and physical exercises in the routine can potentially decrease the prevalence of CMD in women and consequently contribute to their mental and physical well-being.

Common mental disorders in adult women have relevant prevalence rates and associated factors. Reducing the prevalence of CMD is a challenge for public health, because these cases increase the demand and costs of mental health services and other specialties. Common mental disorders not only generate mental suffering, but can also contribute to the worsening of other associated morbidities such as diabetes, heart problems, cancer, and other diseases. Work absenteeism and consequent economic implications are another problem generated by CMD [60].

It is necessary that public policies and programs focused on women’s mental health be able to implement strategies for screening CMD and other disorders. It is important to understand the role of the female sex in CMD, from the in a biological and social point of view, so as to create different strategies for the sexes. Some contexts of health services that serve women should be changed, including professionals trained to develop actions for the prevention and treatment of mental disorders in women, so that management and the community recognize that CMD constitute a public health problem and that women should be evaluated for their mental health in routine primary health care, with actions to minimize the impact of these disorders on women’s health [6].

As for the limitations of the included studies, as they were cross-sectional studies, it is not possible to determine cause-and-effect relations between variables, that is, it is not possible to say whether CMD influence the associated factors or vice versa. Another aspect is that the information in the studies is self-reported and this may generate information bias. Most instruments assess current symptoms, but do not allow the assessment of follow-up of the symptoms. Another limitation found was the lack of identification of confounding factors and of strategies to deal with them in the majority of the studies, indicated in the assessment of the risk of bias.

About the limitation of the present review, articles using different methodologies and samples, different instruments for specific populations, and different cut-off points were included, and this may have caused the wide variability in prevalence rates. Due to this heterogeneity, it was not possible to perform a meta-analysis. Despite the limitations, the present review was carried out with a rigorous methodology by independent authors and reunites important results to guide health policies.

### Conclusion

This review revealed a variation in the prevalence rate of CMD in adult women, with a minimum of 9.6% and maximum of 69.3%. The studies included in this review showed multiple factors associated with CMD, however, only a few stood out, namely, unemployment, low income, low education level, smoking, marital status, experiences of violence, and poor self-rated health. These findings were explained by the different social, economic, demographic conditions and access to health services in the countries.

The results obtained in this review provide essential information for health management and can serve as a basis for reflections on public policies aimed at mental health and women’s health. Based on the knowledge about the factors associated with CMD, policies are needed to create strategies and social support networks to prevent and minimize these illnesses in women. Other types of studies are essential to establish causal relationships of CMD.

## Abbreviations

CIDI, composite international diagnostic interview; CMD, common mental disorders; DSM-V, diagnostic and statistical manual of mental disorders; GHQ-12, general health questionnaire; GPs, Gatekeeper; K6, Kessler 6; MINI, mini-international neuropsychiatric interview; PRISMA, preferred reporting items for systematic reviews and meta-analyses; PRIME-MD, primary care evaluation of mental disorders; PROSPERO, international prospective register of systematic reviews; SRQ-20, self-reporting questionnaire.
