# Supplementary material for: Prevalence and Associated Factors of Common Mental Disorders in Women: A Systematic Review
Source: Public Health Rev. 2021 Aug 23;42:1604234. doi: 10.3389/phrs.2021.1604234 (PMC8419231; doi:10.3389/phrs.2021.1604234)
Supplement: Supplementary file 1 [file DataSheet1.zip › Appendix B.docx]

Appendix B. Excluded articles and reasons for exclusion (n = 54). Prevalence and associated factors of common mental disorders in women: a systematic review, 2020.

| Author, Year | Reason for exclusion |
| --- | --- |
| Stewart et al. 2010 [62] | 4 |
| Kagee et al. 2017 [63] | 4 |
| Smolen et al. 2018 [64] | 1 |
| Chong et al. 2012 [65] | 5 |
| Kagee et al. 2018 [66] | 5 |
| Shaw et al. 1999 [67] | 4 |
| Hanlon et al. 2008 [68] | 1 |
| Velde et al. 2019 [69] | 4 |
| Jenkins et al. 2015 [70] | 2 |
| Neto et al. 2019 [71] | 4 |
| Jacka et al. 2012 [72] | 4 |
| Fahey et al. 2016 [73] | 4 |
| Do Carmo et al. 2018 [74] | 5 |
| Grammatikopoulos et al. 2014 [75] | 5 |
| Rees et al. 2014 [76] | 1 |
| Yu et al. 2016 [77] | 5 |
| Bhui et al. 2014 [78] | 4 |
| Ahmad et al. 2016 [79] | 3 |
| Lima et al. 2006 [80] | 3 |
| Braden et al. 2008 [81] | 4 |
| Brewis et al. 2019 [82] | 2 |
| Bell et al. 2015 [83] | 1 |
| Cunha et al. 2008 [84] | 2 |
| Senicato et al. 2018 [85] | 1 |
| Kallakuri et al. 2018 [86] | 2 |
| Bhui et al. 2001 [87] | 1 |
| Parreira et al. 2017 [88] | 1 |
| Kermode et al. 2010 [89] | 1 |
| Lang et al. 2011 [90] | 1 |
| Kiedjna et al. 2015 [91] | 2 |
| Krueger et al. 2001 [92] | 3 |
| Conway et al. 2013 [93] | 3 |
| Patel et al. 2006 [94] | 2 |
| Puertas et al. 2006 [95] | 5 |
| Levav et al. 20017 [96] | 5 |
| McCrea et al. 2012 [97] | 1 |
| Oleski et al. 2011 [98] | 5 |
| King et al. 2008 [99] | 2 |
| Fahey et al. 2016 [100] | 2 |
| Lahelma et al. 2006 [101] | 2 |
| Said et al. 2013 [102] | 1 |
| Haghighatdoost. 2019 [103] | 3 |
| Thom, 2009 [104] | 2 |
| Bhui et al. 2002 [105] | 3 |
| Myer et al. 2009 [106] | 5 |
| Augusto et al. 2016 [107] | 5 |
| Santos et al. 2019 [108] | 1 |
| Jacka et al. 2011 [109]  Nielsen et al. 2013 [110] | 5 |
|  | 2 |
| Tesfaye et al. 2014 [111] | 5 |
| Patel et al. 1999 [112] | 2 |
| De Moraes et al. 2016 [113] | 5 |
| Meng et al. 2013 [114] | 2 |
| De Silva et al. 2007 [115] | 1 |
| Exclusion criteria: 1 - The abstract did not include the age of the participants and after reading the article, they were outside the age range of the inclusion criteria (n = 14); 2 - The type of study was not clear (n = 12); 3 - The study does not differentiate CMD in men and women (n = 6); 4 - The study focuses more on other subjects (n = 9); 5 – The study does not include factors associated with CMD (n = 13). | |
