# Supplementary material for: Prevalence and Associated Factors of Common Mental Disorders in Women: A Systematic Review
Source: Public Health Rev. 2021 Aug 23;42:1604234. doi: 10.3389/phrs.2021.1604234 (PMC8419231; doi:10.3389/phrs.2021.1604234)
Supplement: Supplementary file 1 [file DataSheet1.zip › Appendix C.docx]

**Appendix C** - Risk of bias of the studies. Quality assessment tool The Joanna Briggs Institute (JBI) Critical Appraisal Checklist for Analytical Cross-Sectional Studies. Prevalence and associated factors of common mental disorders in women: a systematic review, 2020.

| **ITEM** | **Ansseau et al. [14]** | **Audi et al. [15]** | **Borges et al. [6]** | **Campo-arias et al. [21]** | **Cohidon et al. [24]** | **Duran et al. [22]** | **Fortes et al. [19]** | **Ghuloum et al. [31]** | **Girolamo et al. [23]** | **Gjesdal et al. [27]** | **Husain et al. [30]** | **Jansen et al. [20]** | **Jurado et al. [26]** | **Rocha et al. [16]** | **Rocha et al. [17]** | **Santos et al. [18]** | **Skapinakis et al. [25]** | **Soni et al. [28]** | **Menil et al. [29]** |
| --- | --- | --- | --- | --- | --- | --- | --- | --- | --- | --- | --- | --- | --- | --- | --- | --- | --- | --- | --- |
| 1) Were the criteria for inclusion in the sample clearly defined? | U | Y | Y | Y | U | Y | Y | Y | Y | Y | Y | Y | Y | U | Y | Y | U | Y | U |
| 2) Were the study subjects and the setting described in detail? | Y | Y | Y | Y | Y | Y | Y | Y | Y | Y | Y | Y | Y | Y | Y | Y | Y | Y | Y |
| 3) Was the exposure measured in a valid and reliable way? | Y | Y | Y | Y | Y | Y | Y | Y | Y | Y | Y | Y | Y | Y | Y | Y | Y | Y | N |
| 4) Were objective, standard criteria used for measuring the condition? | Y | U | Y | U | Y | Y | Y | Y | Y | Y | Y | Y | Y | Y | Y | U | Y | Y | Y |
| 5) Were confounding factors identified? | U | U | U | N | U | Y | U | U | U | U | U | Y | Y | U | Y | U | U | Y | U |
| 6) Were strategies to deal with confounding factors stated? | N | N | N | N | N | Y | Y | N | Y | U | N | Y | Y | Y | U | N | N | Y | U |
| 7) Were the outcomes measured in a valid and reliable way? | Y | Y | Y | Y | Y | Y | Y | Y | Y | Y | Y | Y | Y | Y | Y | Y | Y | Y | Y |
| 8) Was appropriate statistical analysis used? | Y | Y | Y | Y | Y | Y | Y | Y | Y | Y | Y | Y | Y | Y | y | y | Y | y | Y |
| **Total/Applicable Items** [Not Applicable (NA) items were excluded from the sum] | Moderate 62.5% of Yes | Moderate 62.5% of Yes | Low 75% of Yes | Moderate 62.5% of Yes | Moderate 62.5% of Yes | Low 100% of Yes | Low 75% of Yes | Low 75% of Yes | Low 87.5 of Yes | Low 75% of Yes | Low 75% of Yes | Low 100% of Yes | Low 75% of Yes | Low 75% of Yes | Low 87.5 of Yes | Moderate 62.5% of Yes | Moderate 62.5% of Yes | Low 100% of Yes | Moderate 50% of Yes |
| Abbreviations: Y= Yes; N= No; U= Unclear; NA= Not applicable | | | | | | | | | | | | | | | | | | | |
