# Supplementary material for: Prevalence and Associated Factors of Common Mental Disorders in Women: A Systematic Review
Source: Public Health Rev. 2021 Aug 23;42:1604234. doi: 10.3389/phrs.2021.1604234 (PMC8419231; doi:10.3389/phrs.2021.1604234)
Supplement: Supplementary file 1 [file DataSheet1.zip › Appendix A_.docx]

| PUBMED | SCOPUS | Web of Science | CINAHL | Science direct | Google Scholar | Open Grey |
| --- | --- | --- | --- | --- | --- | --- |
| ("Women"[Mesh] or "Women's Groups”) AND ("Mental Disorders"[Mesh] or “behavior disorders” or “common mental disorders”) | (TITLE-ABS-KEY ("Women" or "Women's Groups”) AND TITLE-ABS-KEY ("Mental Disorders" or “behavior disorders” or “common mental disorders”) | ("Women" or "Women's Groups”) AND Tópico: ("Mental Disorders" or “behavior disorders” or “common mental disorders”) | SU (“Women” or “Women's Groups” ) AND SU ( “Mental Disorders” or “behavior disorders” or “common mental disorders” ) | ("Women" or "Women's Groups”) AND ("Mental Disorders" or “behavior disorders” or “common mental disorders”) | Women AND Mental Disorders | Women AND Mental Disorders |

Appendix A. Database search strategy. Prevalence and associated factors of common mental disorders in women: a systematic review, 2020.
